# Photoelectrochemical Gas–Electrolyte–Solid Phase Boundary for Hydrogen Production From Water Vapor

**DOI:** 10.3389/fchem.2018.00598

**Published:** 2018-12-03

**Authors:** Fumiaki Amano, Ayami Shintani, Hyosuke Mukohara, Young-Min Hwang, Kenyou Tsurui

**Affiliations:** ^1^Department of Chemical and Environmental Engineering, The University of Kitakyushu, Kitakyushu, Japan; ^2^Precursory Research for Embryonic Science and Technology (PRESTO), Japan Science and Technology Agency (JST), Kawaguchi, Japan

**Keywords:** gas-phase water splitting, solar H_2_ production, visible-light-driven photoelectrode, tungsten oxide photoanode, proton exchange membrane

## Abstract

Hydrogen production from humidity in the ambient air reduces the maintenance costs for sustainable solar-driven water splitting. We report a gas-diffusion porous photoelectrode consisting of tungsten trioxide (WO_3_) nanoparticles coated with a proton-conducting polymer electrolyte thin film for visible-light-driven photoelectrochemical water vapor splitting. The gas–electrolyte–solid triple phase boundary enhanced not only the incident photon-to-current conversion efficiency (IPCE) of the WO_3_ photoanode but also the Faraday efficiency (FE) of oxygen evolution in the gas-phase water oxidation process. The IPCE was 7.5% at an applied voltage of 1.2 V under 453 nm blue light irradiation. The FE of hydrogen evolution in the proton exchange membrane photoelectrochemical cell was close to 100%, and the produced hydrogen was separated from the photoanode reaction by the membrane. A comparison of the gas-phase photoelectrochemical reaction with that in liquid-phase aqueous media confirmed the importance of the triple phase boundary for realizing water vapor splitting.

## Introduction

Large-scale deployment of photocatalytic and photoelectrochemical (PEC) water-splitting technologies, accompanied by fuel cells and energy carrier technologies, will allow the realization of the hydrogen (H_2_) economy (Maeda and Domen, 2010). Separation of H_2_ and oxygen (O_2_) is easily achieved in PEC systems that use solid polymer electrolyte membranes (Pinaud et al., 2013; McKone et al., 2014). Laboratory-scale PEC studies are usually performed in aqueous electrolyte solutions using purified water without contamination. However, in practice, water resources are an issue for solar H_2_ production. Stable supply of water might be problematic for large-scale solar H_2_ production owing to limited rainfall in areas with low-cost land and abundant solar radiation such as deserts (Kumari et al., 2016). Another possible feedstock is seawater, but its use requires purification to avoid problems such as corrosion, poisoning, fouling, and byproduct formation. In contrast, gaseous water has been proposed as an attractive alternative to liquid water because an inexhaustible supply of water vapor from the ambient humid air is available at sea with high relative humidity (~80%) (Kumari et al., 2016). Gas-phase operations can significantly decrease maintenance costs because the natural convection of air can be used to feed the water vapor and systems to purify and pump liquid water are not required (Rongé et al., 2014; Modestino et al., 2015).

Gas-phase water splitting by all-solid PEC systems has been studied using proton exchange membranes (PEMs) as a solid polymer electrolyte and n-type semiconductor electrodes as a photoanode (Georgieva et al., 2009, 2010; Iwu et al., 2013; Rong et al., 2013; Tsui et al., 2013; Rongé et al., 2014; Stoll et al., 2016, 2017; Verbruggen et al., 2017). PEC water oxidation is induced by photogenerated holes on the photoanode, which is in contact with the membrane, whereas the H_2_ evolution reaction occurs on the cathode, which is located on the opposite side of the membrane. The PEM-based photoelectrolyzer (PEM-PEC cell) can be operated using gas-phase reactants such as water vapor and volatile organic compounds in air. However, the photon-to-current conversion efficiencies of the photoanodes are frequently low owing to the difficulties associated with designing electrodes for “gas–solid” PEC systems in contrast to those for conventional “liquid–solid” systems in aqueous electrolytes. The photoelectrode for a gas-phase reaction should exhibit gas diffusion properties for mass transport and proton conductivity for ion transport toward the membrane. It has been proposed that high-surface-area photoanodes need to be covered with a polymer electrolyte thin film to maximize the gas–electrolyte–solid phase contact area (Spurgeon and Lewis, 2011; Xiang et al., 2016). However, the effect of such a triple phase boundary has not been elucidated for gas–solid PEC systems. In addition, most studies have focused on TiO_2_-based photoanodes under UV irradiation, and there are only a few reports on visible-light-responsive photoelectrodes for gas-phase reactions (Georgieva et al., 2009; Stoll et al., 2017). The use of narrow band gap semiconductors is necessary to achieve high solar-to-hydrogen efficiency because the number of photons is limited in the UV range. The maximum solar-to-hydrogen efficiency is only 1.7% even if all the UV light with wavelength shorter than 400 nm is utilized for water splitting reaction under AM1.5G spectrum (Abe, 2010; Hisatomi et al., 2015).

The aim of this study was to develop an all-solid PEC system with a triple phase boundary for water vapor splitting under visible-light irradiation. We selected tungsten trioxide (WO_3_) as a blue-light responsive semiconductor with a narrow band gap of 2.6–2.7 eV (Amano et al., 2013). High-surface-area WO_3_ nanoparticles were deposited on porous titanium microfiber felt (WO_3_/Ti fiber), which was then covered with a perfluorosulfonic acid ionomer electrolyte thin film to improve its proton transport properties. The perfluorosulfonate ionomer, DuPont Nafion®, exhibits good proton conductivity at room temperature in hydrous conditions (Kusoglu and Weber, 2017). The Nafion ionomer thin film also has high chemical stability, moderate gas permeability, and moisture absorbency, which allows it to capture humidity in the gas phase (Modestino et al., 2015). We fabricated a membrane electrode assembly (MEA) by hot pressing the WO_3_/Ti-fiber electrode onto a PEM with a platinum catalyst film on the opposite side. The effect of the ionomer thin film loading on the PEC performances of the WO_3_/Ti fiber electrode was investigated in both humidified argon and liquid water using two-electrode and conventional three-electrode configurations.

## Materials and methods

### Preparation of WO_3_ gas diffusion electrode

Sintered Ti microfiber felt (thickness: 0.1 mm, Nikko Techno, Japan) was used as a macroporous conductive substrate to prepare a gas diffusion electrode composed of WO_3_ nanoparticles (Homura et al., 2014; Amano et al., 2017). Ammonium metatungstate hydrate (20.4 g, Nippon Inorganic Colour & Chemical, Japan) and PEG 20,000 (10.0 g, Wako Pure Chemical, Japan) were dissolved in deionized water (40.0 g) as a solution for dip coating of the Ti microfiber felt. The dip coating process was performed three times. The precursor-coated Ti microfiber felt was dried at 353 K and calcined in air at 923 K for 2 h to achieve crystallization of WO_3_. If required, the obtained WO_3_/Ti fiber electrode was treated with a Nafion® perfluorosulfonic acid (PFSA) ionomer dispersion (5 wt% in mixture of lower aliphatic alcohols and water, contains 45% water, Sigma-Aldrich Japan, Japan) as a proton-conducting polymer. The dispersion was dropped on the electrode (10 μL cm^−2^), and the wetted electrode was dried at 353 K. The loading amount of the Nafion ionomer was ~0.6 mg cm^−2^.

### Preparation of membrane electrode assembly

A carbon black-supported platinum nanoparticles (Pt/CB) was used as a cathode catalyst for H_2_ evolution. A cathode catalyst ink was prepared by ultrasonication of a mixture of a Pt/CB (TEC10E50E, Pt loading: 46.6 wt%, Tanaka Kikinzoku Kogyo, Japan) and the Nafion ionomer dispersion. The weight ratio of the Nafion ionomer to Pt was adjusted to 1.0, and the loading amount of platinum was ~0.1 mg cm^−2^. The ionomer-mixed Pt/CB film and the WO_3_/Ti fiber electrode were hot pressed onto a Nafion membrane N117 (thickness: 183 μm, DuPont, USA) at 15 kN and 413 K. The composition of the MEA was “WO_3_/Ti fiber | Nafion membrane | ionomer-mixed Pt/CB.”

### Characterization

Scanning electron microscopy (SEM) observation and energy dispersive X-ray spectroscopy (EDS) analysis were performed on a JSM-7800F microscope (JEOL, Japan). The electrode was directly deposited on carbon tape. High-magnification SEM observation was performed on S-5200 microscope (Hitachi, Japan) with an operating voltage of 5 kV. Before the high-magnification observation, the sample deposited on carbon tape was coated with gold using an E-1030 ion sputter coater (Hitachi, Japan).

Nitrogen adsorption isotherms were recorded at 77 K in the relative pressure range between 0.05 and 0.30 with a BELSORP-mini system (MicrotracBEL, Japan). Before the measurements, the electrode was outgassed at 473 K for 2 h. The Brunauer–Emmett–Teller (BET) equation was used to calculate the surface area of WO_3_/Ti fiber electrode. The BET specific surface area of the WO_3_ particles was estimated from the measured surface area of the electrode and the loading amount of the WO_3_ particles. The BET specific surface area of Ti microfibers was smaller than the measurement limit.

### Photoelectrochemical measurements

**Figure 1:**
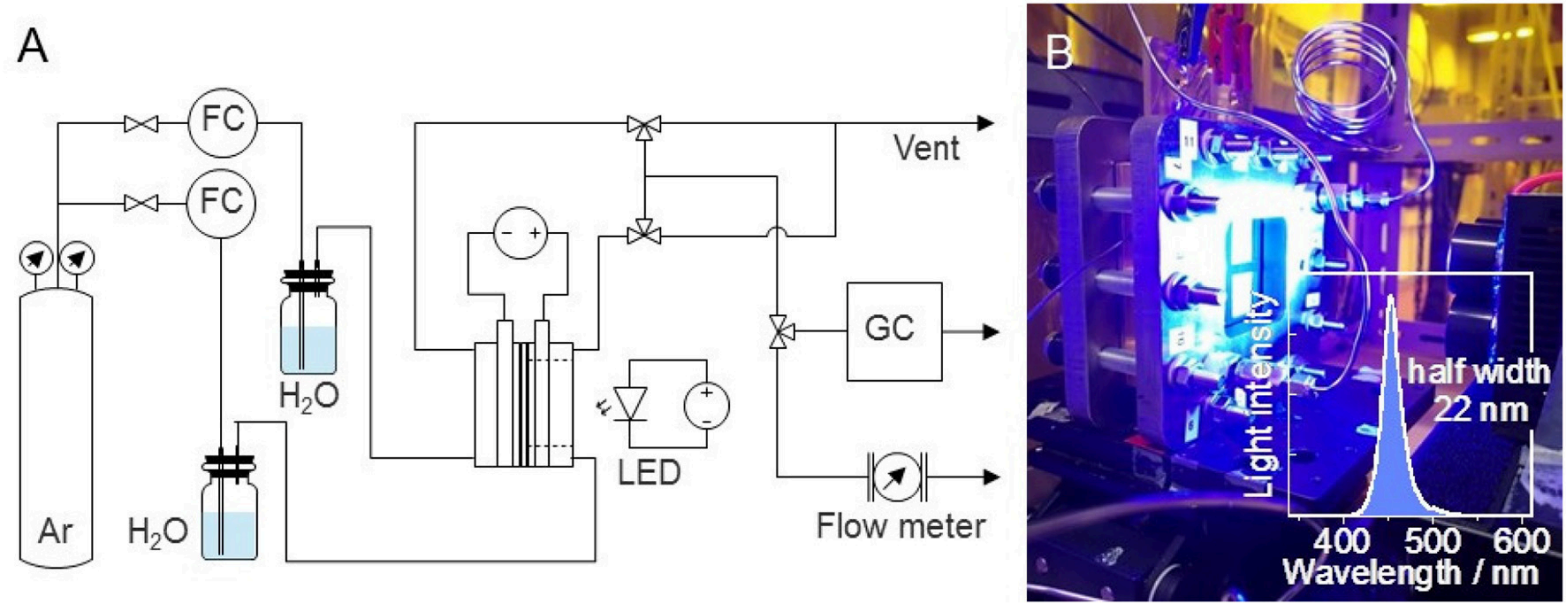
Process flow for photoelectrolysis of water vapor (FC, flow controller; LED, light-emitting diode; GC, gas chromatograph). **(B)** Photograph of the photoelectrolyzer and blue LED (inset: spectrum of the blue light, centered at 453 nm).

The PEC reaction was conducted at room temperature (298 K) and atmospheric pressure (0.1 MPa). The gas-phase water splitting reaction was performed using a dual compartment stainless-steel PEC reactor with an optical window, as shown in Figure 1. The compartments were separated by the Nafion membrane. Water vapor (3 vol%) was introduced into each compartment by passing argon at a flow rate of 20 mL min^−1^ through a bubbler filled with deionized water. The electrode area was 25 cm^2^, but the light irradiation area was 16 cm^2^ owing to the presence of a gold-coated copper plate as the current collector. Photoirradiation was performed using 3 W blue light-emitting diode lamps. The emission was centered at a wavelength (λ) of 453 nm with a full width at half maximum of 22 nm. The optical power was measured to be ~7 mW cm^−2^. A 300 W xenon lamp with bandpass filters (bandwidth ~10 nm) was used to obtain the action spectrum for the incident photon-to-current conversion efficiency (IPCE), which is the ratio of the number of electric charges to the number of incident photons, as shown in equation (1).

(1) $$\begin{array}{r} \text{IPCE}=\left(i_{\text{photo}}\times 1240/\lambda \right)/I_{0} \end{array}$$

where *i*_photo_ is the steady-state photocurrent density [mA cm^−2^], 1240/λ is the monochromatic photon energy [eV], and *I*_0_ is the power of the incident monochromatic light [mW cm^−2^]. The Faraday efficiency (FE), also known as the current efficiency, was calculated from the ratio of the electric current required for the formation of a product to the total electric current, as shown in equation (2).

(2) $$\begin{array}{r} \text{FE}=\left({\text{n}}_{\text{e}}\times \text{F}\times \text{r}\left(\text{x}\right)\right)/{\text{i}}_{\text{photo}} \end{array}$$

where *n*_e_ is the number of electrons required to form a product, *F* is the Faraday constant, and *r*(*x*) is the formation rate of product *x*. The amounts of evolved O_2_ and H_2_ were analyzed using an online gas chromatograph equipped with a thermal conductivity detector and an MS-5A column with argon as the carrier gas. The *n*_e_ values of H_2_ and O_2_ are 2 and 4, respectively.

An H-type dual compartment glass reactor was used for the PEC measurements of small area electrodes (light irradiation area: 1 cm^2^). The compartment separated by the Nafion membrane was filled with 3 vol% H_2_O vapor in argon or an aqueous electrolyte of 0.1 mol L^−1^ sulfuric acid (pH = 1). A silver/silver chloride (Ag/AgCl) reference electrode (+195 mV vs. the standard hydrogen electrode) was used in the three-electrode configuration. The Nernst equation [equation (3)] can be used to convert the electrode potential vs. Ag/AgCl (*E*_Ag/AgCl_) to the potential vs. the reversible hydrogen electrode (*E*_RHE_).

(3) $$\begin{array}{r} {\text{E}}_{\text{RHE}}={\text{E}}_{\text{Ag}/\text{AgCl}}+0.059\text{pH}+0.195 \end{array}$$

## Results

### Preparation of the ionomer-coated WO_3_ photoelectrode

**Figure 2:**
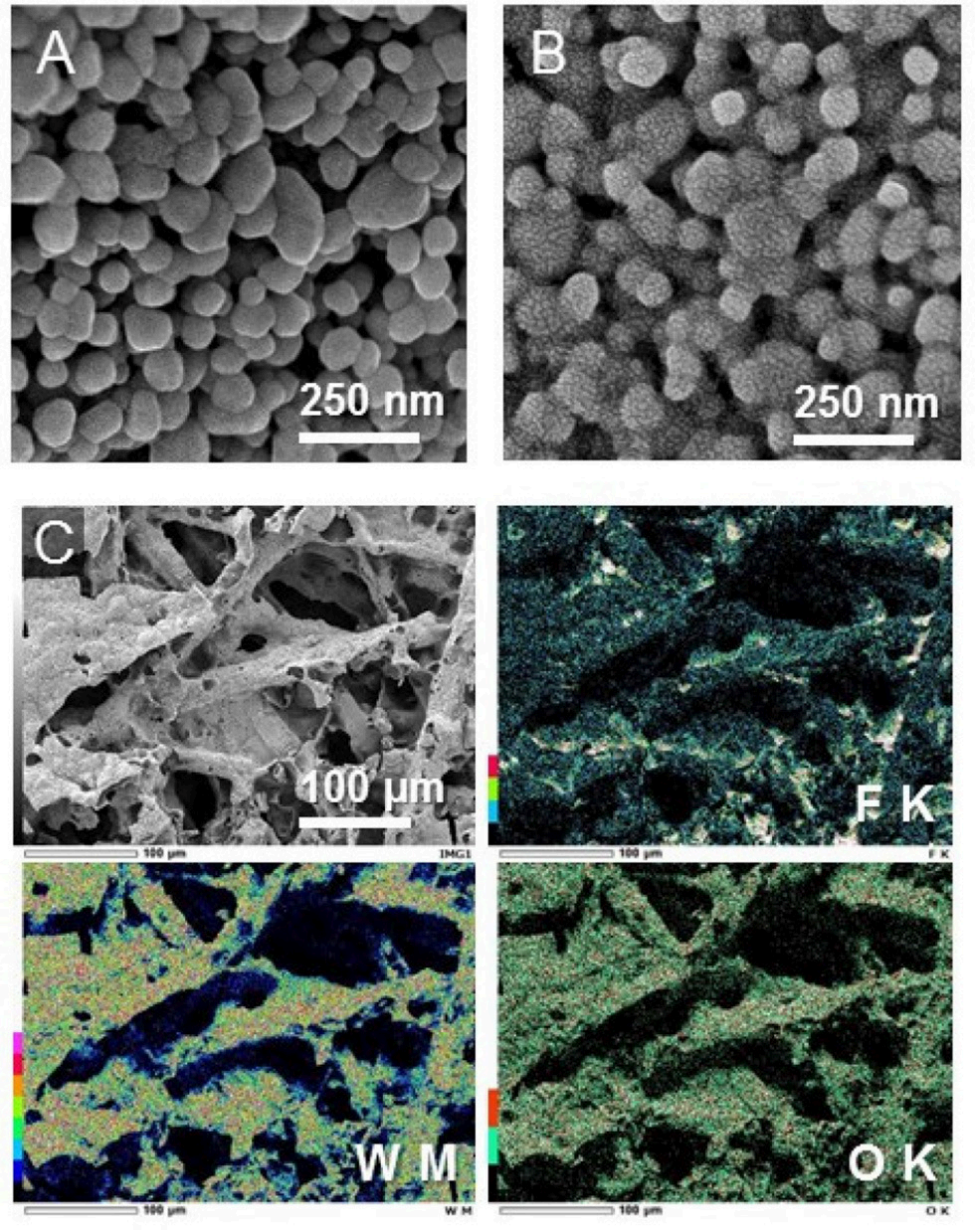
SEM images of **(A)** the bare WO_3_/Ti fiber electrode and **(B)** the ionomer-coated WO_3_/Ti fiber electrode. The images were obtained after sputtering with gold. **(C)** EDS mapping images for F, W, and O elements of the ionomer-coated WO_3_/Ti fiber electrode.

To allow for gas diffusion and ion transport, the PEM-based PEC cell for gaseous reactants requires a three-dimensional porous electrode rather than a conventional planar dense electrode. We used a titanium microfiber felt that has a high porosity (67%) owing to its three-dimensional fibrous structures with diameters of 20 μm (Amano et al., 2017). The specific surface area of the Ti microfiber felt with a thickness of 0.1 mm was ~450 cm^2^ g^−1^, which is 90 times larger than that of a conventional two-dimensional Ti sheet with a thickness of 1.0 mm (5 cm^2^ g^−1^). The formation of a highly crystalline monoclinic WO_3_ phase was confirmed by X-ray diffraction and Raman spectroscopy (Amano et al., submitted). The loading amount of WO_3_ was 13 mg cm^−2^, which corresponds to 27 wt% in the WO_3_/Ti fiber electrode. SEM images of the WO_3_/Ti fiber after gold sputtering (Figure 2A) revealed that monoclinic WO_3_ formed nanoparticles with particle diameters of ~100 nm. The BET specific surface area of the WO_3_ particles was measured to be 7.5 m^2^ g^−1^, which corresponds to the average diameter of 110 nm assuming that each particle was a sphere. The estimated particle size was similar to that of the WO_3_ particles observed in SEM images. The PEC performance for water oxidation was tested in a conventional aqueous electrolyte solution (phosphate buffer, pH = 2.2). The performance of the WO_3_/Ti fiber electrode was superior to that of a WO_3_ particle electrode obtained by deposition of WO_3_ nanoparticles on a two-dimensional substrate such as transparent conductive oxide-coated glass and a titanium sheet (Amano et al., 2017). The IPCE of the WO_3_/Ti fiber electrode was ~60% at 1.0 V vs. Ag/AgCl under UV irradiation, and the FE of O_2_ evolution was higher than 70%.

A Nafion ionomer dispersion was cast on the high-surface-area WO_3_ nanoparticle electrode. Figure 2B shows a high-magnification SEM image of the WO_3_/Ti fiber electrode after gold sputtering. The sputtered gold nanoparticles were clearly observed in the case of the ionomer-coated WO_3_/Ti fiber, suggesting that the Nafion ionomer thin film induced the heterogeneous nucleation of the gold nanoparticles. This phenomenon would be a useful method to visualize the Nafion ionomer thin film in SEM observation. We found that the thin film covered the WO_3_ particles, with some of the ionomer aggregated at the grain boundary. The thickness of the ionomer thin film was estimated to be ~3 nm by using the specific gravity of the Nafion membrane (1.98) assuming that the thin layer uniformly covers the surface of the WO_3_ nanoparticles. EDS elemental analysis confirmed an increase in the content of carbon and fluorine after ionomer coating. The fluorine mapping shows that the ionomer was dispersed on the macroporous electrode, with some of the ionomer segregated in the void spaces of the Ti microfibers (Figure 2C). The ionomer-coated WO_3_/Ti fiber photoanode (5 × 5 cm^2^) and an ionomer-mixed Pt/CB cathode (5 × 5 cm^2^) were pressed on both sides of a Nafion membrane (8 × 8 cm^2^) to fabricate an MEA for a large stainless-steel PEC reactor (irradiated area: 16 cm^2^).

### Gas-phase water splitting in the PEM-PEC reactor

**Figure 3:**
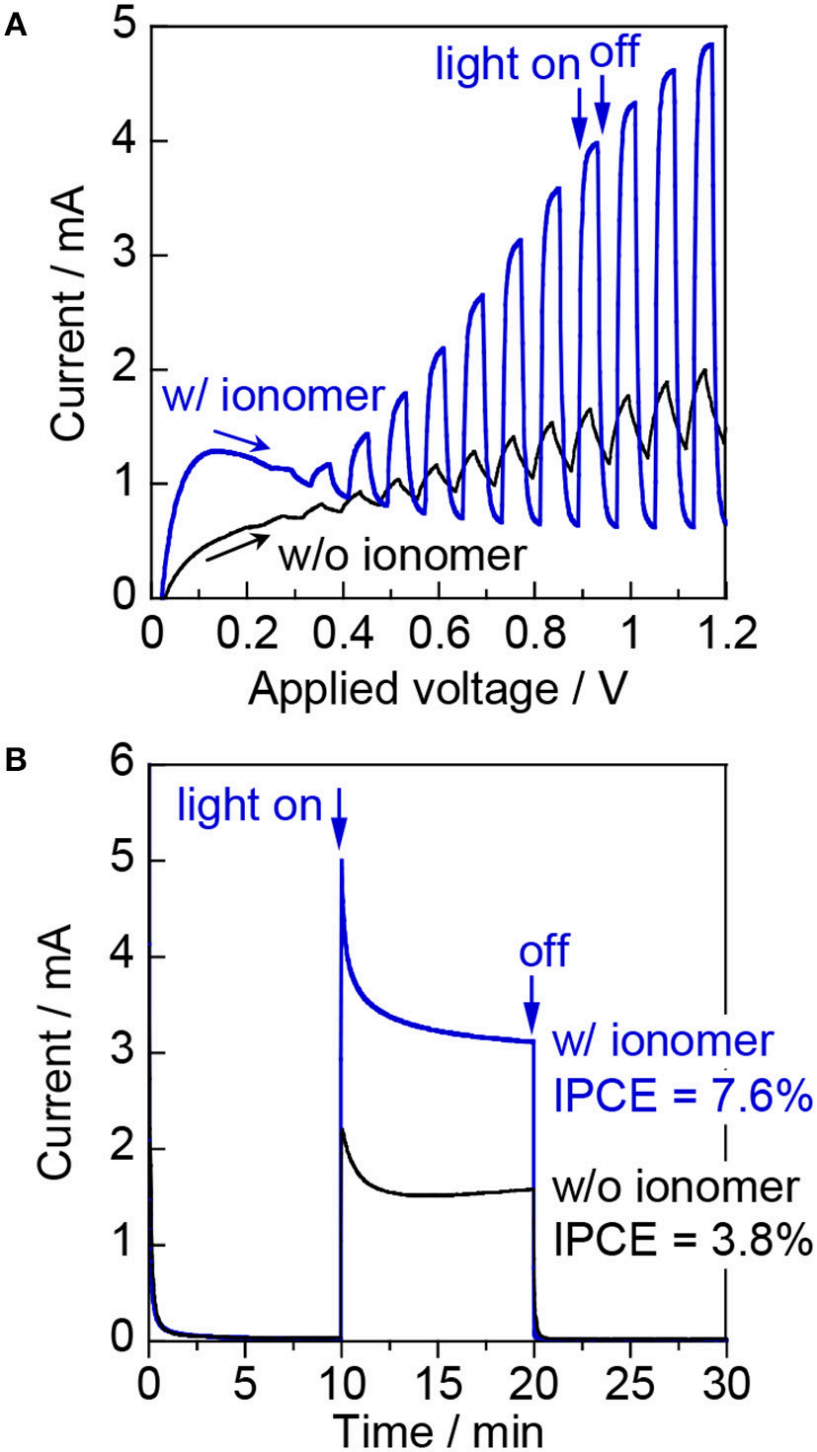
**(A)** Linear sweep voltammetry curves at a scan rate of 20 mV s^−1^ under chopped irradiation (2 s intervals) and **(B)** current–time curves at an applied voltage of 1.2 V for WO_3_/Ti fiber electrodes with and without the Nafion ionomer coating. Reaction conditions: 453 nm blue light, irradiance: 6.8 mW cm^−2^, photoirradiation area: 16 cm^2^, Ar/H_2_O = 97/3, flow rate to each electrode: 20 mL min^−1^.

Figure 3A shows the photoresponse of the WO_3_/Ti fiber photoanode with and without the ionomer in an argon flow with 3 vol% H_2_O vapor (relative humidity: ~90%) under chopped visible-light irradiation (λ = 453 nm). The two compartments of the large PEM-PEC cell were pre-purged with water vapor for more than 1 h. The applied voltage corresponds to the potential difference between the WO_3_/Ti fiber photoanode and the ionomer-mixed Pt/CB cathode. The WO_3_/Ti fiber electrode without the ionomer coating exhibited small photocurrent and a slow photoresponse during the gas-phase PEC measurements. The photocurrent was significantly decreased compared with that observed for the PEC performance test in a conventional aqueous electrolyte solution. In contrast, the ionomer-coated WO_3_/Ti fiber electrode showed an increased photocurrent response in the gas-phase PEC measurements. The photocurrent at applied voltages higher than 0.2 V quickly increased when the UV light was turned on, and quickly decayed to the dark current level when the light was turned off. Thus, we found that the photoresponse of the WO_3_/Ti fiber photoanode was accelerated by the Nafion ionomer coating, indicating that the electron transfer from water to WO_3_ was accelerated. Figure 3B shows the time course of the photocurrent at 1.2 V. A Faraday current was not observed at steady state in the dark. The IPCE at 1.2 V was 7.6% for the ionomer-coated WO_3_/Ti fiber photoanode, but the IPCE was only 3.8% in the absence of the ionomer coating. Moreover, an anodic current owing to water vapor oxidation was observed only under photoirradiation. Thus, the Nafion ionomer coating enhanced the steady-state photocurrent of the WO_3_/Ti fiber electrode.

**Figure 4:**
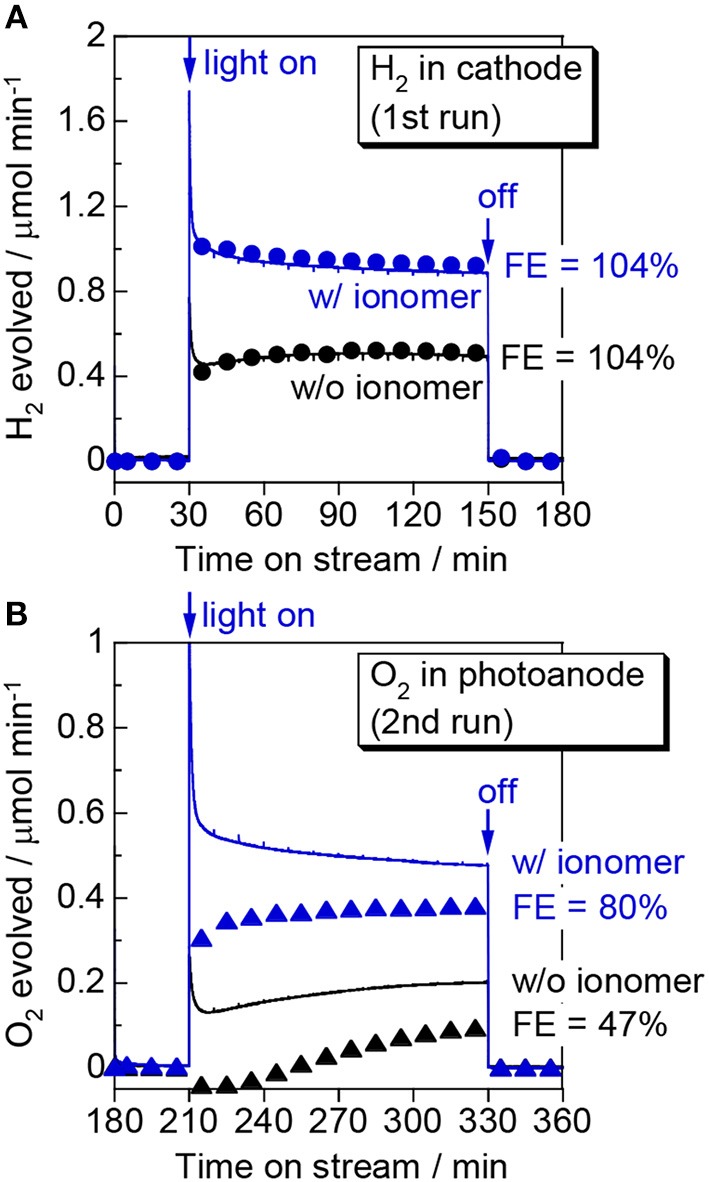
Time course of **(A)** H_2_ evolution in the first run and **(B)** O_2_ evolution in the second run during gas-phase water splitting over WO_3_/Ti fiber photoelectrodes with and without a Nafion ionomer coating. We measured the H_2_ evolved in the cathode compartment in the first run and the O_2_ evolved in the photoanode compartment in the second run. The symbols show the product formation rate determined by gas chromatography and the curves show the electron flow passing through the outer circuit (e^−^/2 for H_2_ and e^−^/4 for O_2_). Reaction conditions: 453 nm blue light, irradiance: 6.8 mW cm^−2^, photoirradiation area: 16 cm^2^, applied voltage: 1.2 V, Ar/H_2_O = 97/3, flow rate to each electrode: 20 mL min^−1^, temperature: 298 K, pressure: 0.1 MPa.

Figure 4 shows the outcome of the visible-light-induced gas-phase water-splitting reaction in the PEM-PEC reactor. The applied voltage was set to 1.2 V, which is less than the thermodynamic minimum voltage required for water electrolysis at room temperature (1.23 V). The reaction was repeated twice to check the evolved gasses in the photoanode and cathode compartments individually. In the first run, we analyzed the gas evolved from the cathode compartment. We detected continuous H_2_ formation over the ionomer-mixed Pt/CB catalyst cathode when the photoanode was irradiated with blue light. The H_2_ production rate in the cathode compartment was consistent with half of the electron flow in the outer circuit (e^−^/2). The FE of H_2_ evolution was close to 100%, assuming a two-electron reaction (2H^+^ + 2e^−^ → H_2_). This result indicates that the photoexcited electrons in the conduction band of WO_3_ are transported via the outer circuit to the counter electrode to reduce protons and evolve H_2_. The visible-light-induced H_2_ production rate was ~1.0 μmol min^−1^.

In the second run, we analyzed the gas in the photoanode compartment. Although we confirmed that O_2_ evolution occurred under photoirradiation, the O_2_ formation rate was less than a quarter of the electron flow (e^−^/4). It should be noted that the formation rate shows a net increase of O_2_ because a small amount of O_2_ was mixed in the flow from the ambient air. When the light was turned on, we observed an initial decrease in the O_2_ concentration for the bare WO_3_/Ti fiber photoanode, likely because the O_2_ contaminant was consumed by a process such as photoabsorption under photoirradiation. The O_2_ evolution rate gradually increased with the PEC reaction, but the FE of O_2_ evolution was only 47%, even just before turning off the light. As the FE was calculated by assuming that four electrons are required for the O_2_ evolution reaction, an FE of <100% indicates the presence of byproducts such as hydrogen peroxide in the photoanode compartment. The formation of hydrogen peroxide via a reaction requiring two electrons was reported for a PEC reaction over a WO_3_ photoanode in an aqueous electrolyte (Santato et al., 2001). In contrast, the Nafion ionomer coating significantly enhanced the FE of O_2_ evolution up to 80%. This result suggests that the proton-conducting thin film promoted the four-electron water oxidation reaction to evolve O_2_. The visible-light-induced O_2_ production rate with this electrode was ~0.4 μmol min^−1^ at an IPCE of 7.5% at 1.2 V.

**Figure 5:**
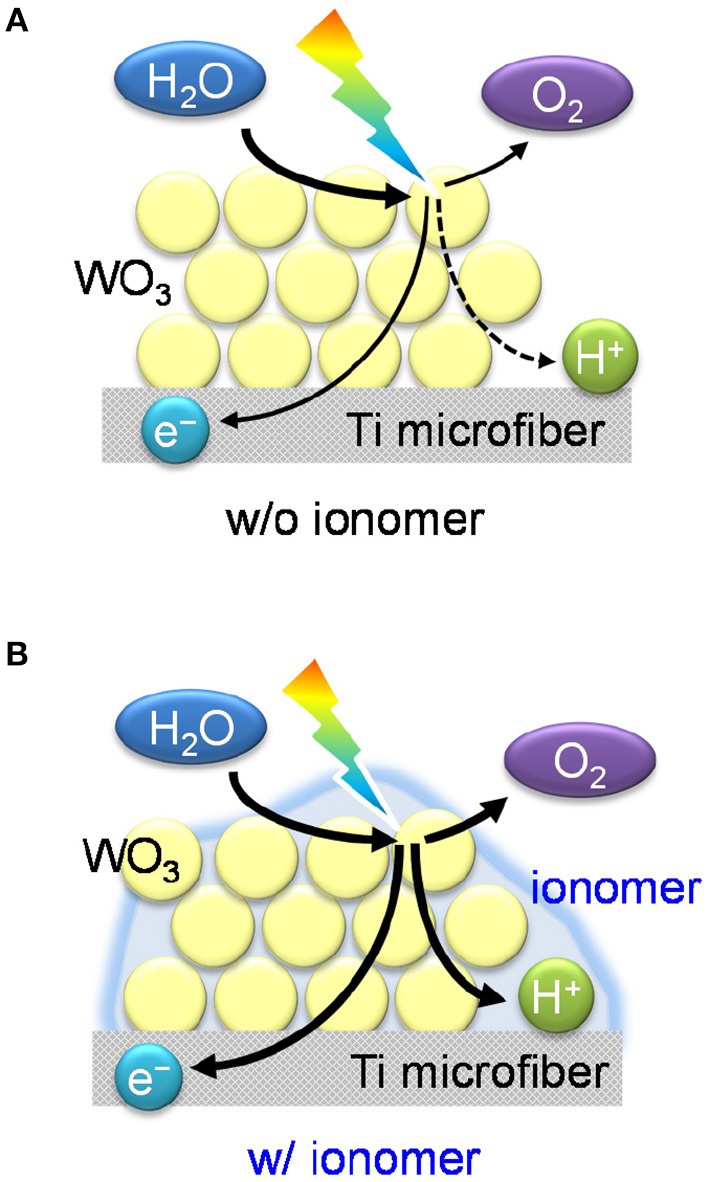
Schematic illustrations of **(A)** the interfaces of the WO_3_/Ti fiber electrode without the ionomer coating and **(B)** the photoelectrochemical triple phase boundary of the ionomer-coated WO_3_/Ti fiber electrode.

Figure 5 shows a proposed schematic mechanism for water vapor oxidation over the ionomer-coated WO_3_/Ti fiber photoanode. The O_2_ evolution reaction (2H_2_O → O_2_ + 4H^+^ + 4e^−^) is recognized as a proton-coupled electron transfer process (Surendranath et al., 2010; Warren et al., 2010). Photocatalysis processes over TiO_2_ and ZnO have also been confirmed as proton-coupled electron transfer reactions (Schrauben et al., 2012). The photogenerated holes in the valence band induce four-electron oxidation of water to evolve O_2_. The concerted transfer process enhances the reaction rate because the transfer of multiple electrons and protons at the same time avoids the formation of the high-energy intermediates obtained during stepwise reactions. However, proton transfer at the gas–solid interface will be difficult in the absence of an aqueous electrolyte, which acts as an ion conductor. Therefore, the proton-coupled electron transfer is the rate-determining step of water vapor oxidation over the bare WO_3_/Ti fiber electrode. However, we found that the proton-conducting ionomer thin film promotes the gas-phase PEC reaction over the WO_3_/Ti fiber electrode. These results clearly indicate the importance of the gas–electrolyte–solid triple phase boundary for the PEC water vapor splitting reaction.

### PEC measurements in the three-electrode configuration

**Figure 6:**
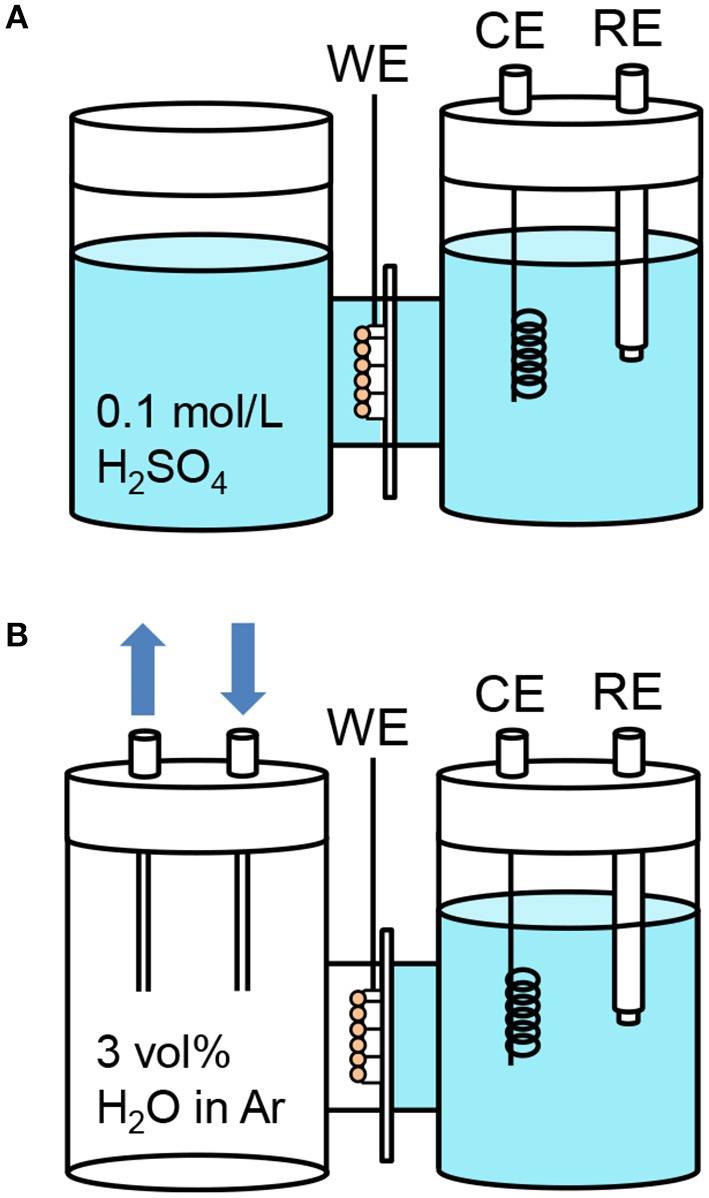
Schematic illustrations of the H-type dual compartment glass reactors used for testing the PEC performance of **(A)** “liquid | solid | liquid” interfaces and **(B)** “gas | solid | liquid” interfaces in the three-electrode configuration. (WE: 1 cm^2^ working electrode pressed on a Nafion membrane, CE: Pt counter electrode, RE, Ag/AgCl reference electrode). The gas flow was argon with 3 vol% water vapor. The aqueous electrolyte was 0.1 mol L^−1^ sulfuric acid (pH = 1).

We aimed to investigate the role of the triple phase boundary on the gas-phase PEC reactions. A small H-type glass cell (irradiated area: 1.0 cm^2^) was used to compare the PEC performance under different conditions. Figure 6 shows the setup for the PEC measurements in the three-electrode configuration using a platinum wire counter electrode and a Ag/AgCl reference electrode. The two compartments were separated by a Nafion membrane. We filled the cathode compartment with an aqueous electrolyte to maintain electrical neutrality between the membrane, the counter electrode, and the reference electrode. The WO_3_/Ti fiber photoanode, which was in contact with the Nafion membrane, was exposed to the other compartment. We investigated two different photoanode conditions. In the first, the photoanode was immersed in an aqueous electrolyte (Figure 6A), whereas in the second, the photoanode was exposed to an argon flow with 3 vol% water vapor (Figure 6B). These setups are denoted as “liquid | solid | liquid” and “gas | solid | liquid” interfaces, respectively.

**Figure 7:**
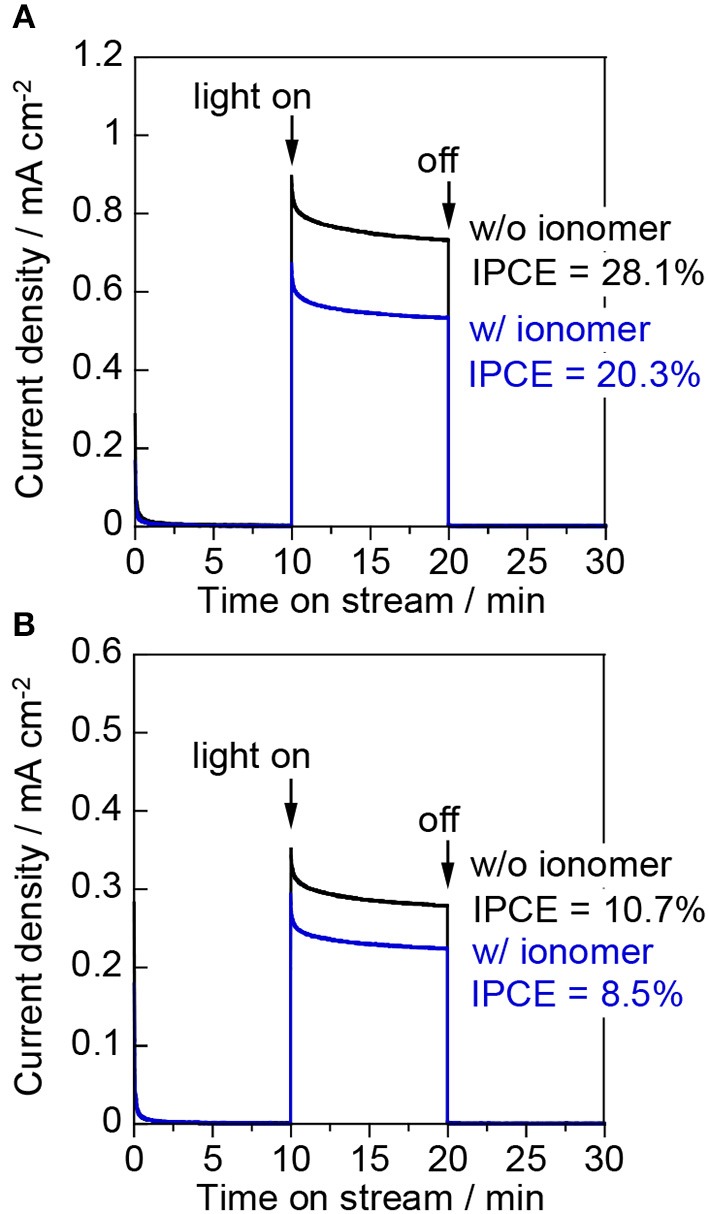
Current–time curves of **(A)** the “liquid | solid | liquid” interfaces and **(B)** the “gas | solid | liquid” interfaces at an applied potential of 1.0 V vs. Ag/AgCl in a three-electrode configuration. The working electrodes were WO_3_/Ti fiber electrodes with and without a Nafion ionomer coating. The irradiation source was 453 nm blue light.

Figure 7A shows the photocurrent density at 1.0 V vs. Ag/AgCl for the “liquid | solid | liquid” interfaces. The photocurrent exhibited by the bare electrode was higher than that of the ionomer-coated electrode during the liquid-phase water splitting reaction. This observation indicates that the Nafion ionomer coating retards the oxidation of water, probably by interrupting water adsorption and/or O_2_ desorption on the WO_3_ surface. Figure 7B shows the photocurrent density at 1.0 V vs. Ag/AgCl for the “gas | solid | liquid” interfaces. The IPCE decreased from 28.1 to 10.7% when the photoanode cell was changed from the liquid phase to the two-phase environment. This decrease indicates that the penetration of the aqueous electrolyte into the interconnected mesopores of the WO_3_ nanoparticles is very important for enhancing the proton-coupled electron transfer process during water oxidation. The photocurrent of the bare electrode decreased more than that of the ionomer-coated electrode when the liquid phase was changed for the two-phase environment. However, the photocurrent of the bare electrode was still higher than that of the ionomer-coated electrode. This is because the Nafion membrane remained fully hydrated when one part of it was in contact with the aqueous electrolyte in the other compartment. Therefore, the bare WO_3_ electrode was also coated with a thin layer of the aqueous electrolyte because it was in contact with the wetted Nafion membrane.

### PEC measurements in the two-electrode configuration

**Figure 8:**
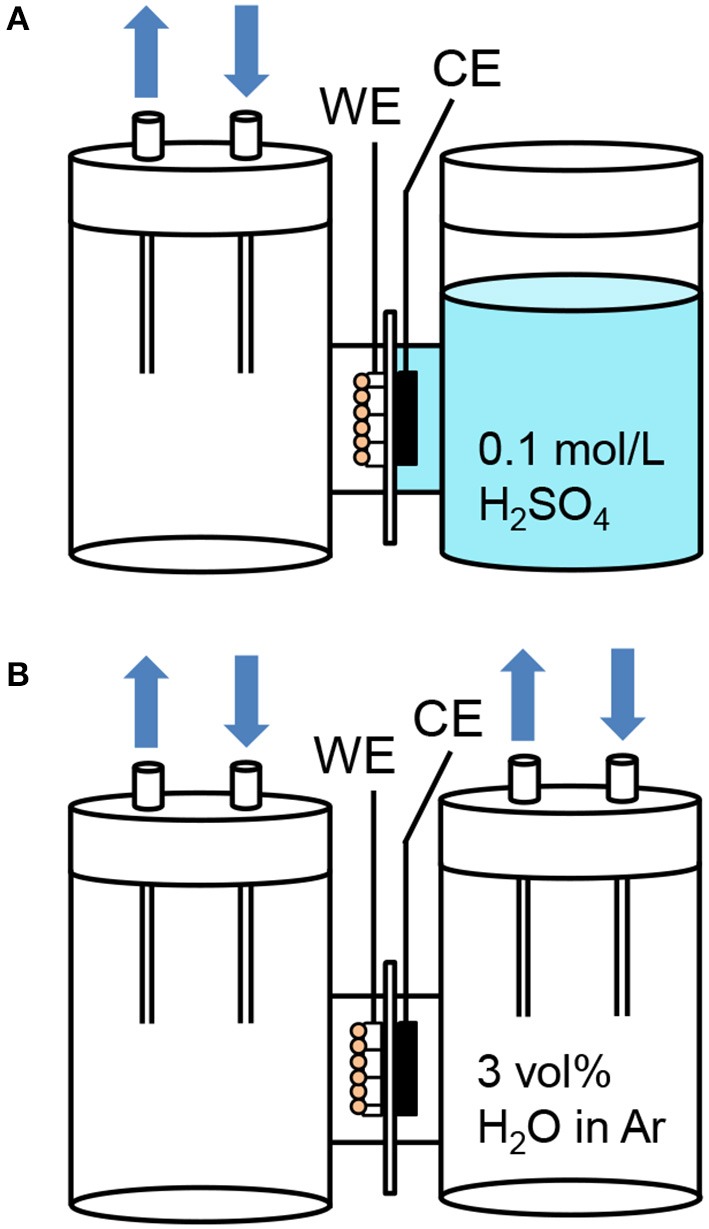
Schematic illustrations of the H-type dual compartment glass reactors using for testing the PEC performance of **(A)** “gas | solid | liquid” interfaces and **(B)** “gas | solid | gas” interfaces in the two-electrode configuration. (WE: 1 cm^2^ working electrode pressed on a Nafion membrane, CE: ionomer-mixed Pt/CB electrode on the opposite side of the membrane).

We further investigated the PEC performance of the WO_3_/Ti fiber photoanodes in the two-electrode configuration to compare two different cathode conditions as shown in Figure 8. The ionomer-mixed Pt/CB film was used as a counter electrode in place of the platinum wire in the three-electrode configuration. The WO_3_/Ti fiber and the ionomer-mixed Pt/CB film were in contact with a Nafion membrane. For the “gas | solid | liquid” interfaces, the WO_3_/Ti fiber photoanode was exposed to the gas phase and the ionomer-mixed Pt/CB cathode was immersed in an aqueous electrolyte (Figure 8A). For the “gas | solid | gas” interfaces, both the photoanode and the cathode were exposed to the gas phase (Figure 8B). The electrode potential of 1.0 V vs. Ag/AgCl at pH = 1 corresponds to 1.25 V vs. RHE. Therefore, we set the applied voltage to 1.20 V in the two-electrode configuration.

**Figure 9:**
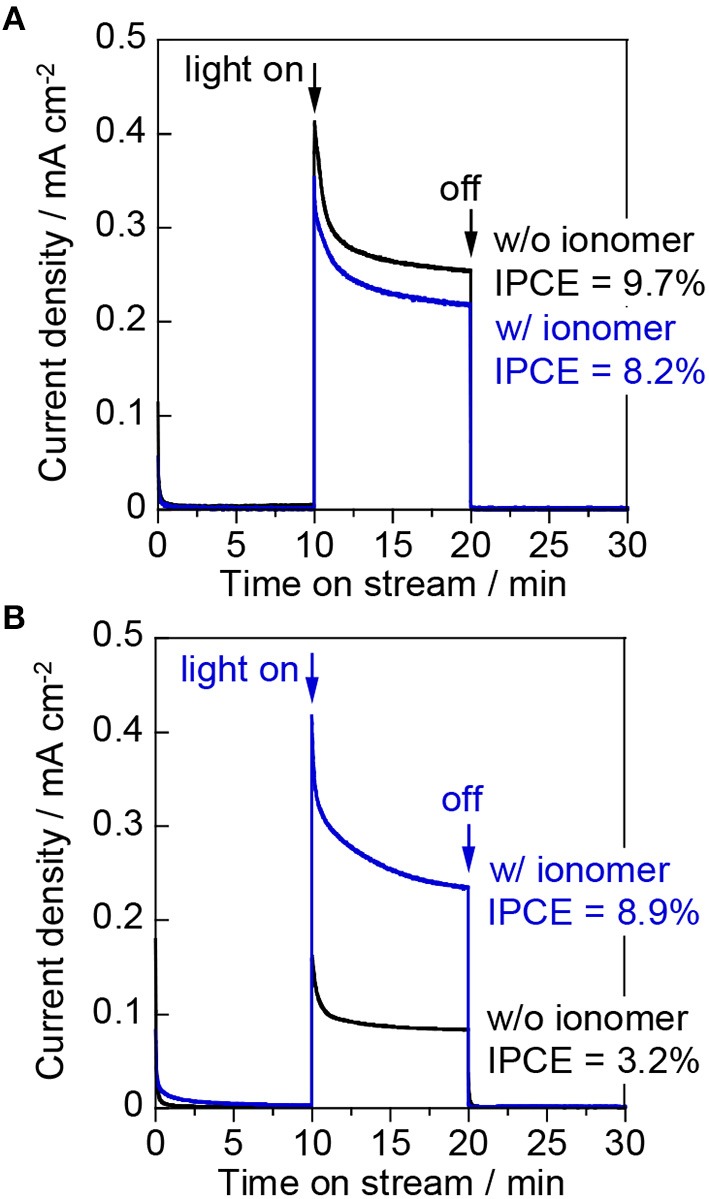
Current–time curves of **(A)** the “gas | solid | liquid” interfaces and **(B)** the “gas | solid | gas” interfaces at an applied voltage of 1.2 V in the two-electrode configuration. The working electrodes were WO_3_/Ti fiber electrodes with and without a Nafion ionomer coating. The irradiation source was 453 nm blue light.

Figure 9A shows the results for the “gas | solid | liquid” interfaces in the two-electrode system. The photocurrent densities at an applied voltage of 1.2 V were similar to those at 1.0 V vs. Ag/AgCl in the three-electrode configuration. This result indicates that the cathodic polarization distributed from the applied voltage was small on the ionomer-mixed Pt/CB catalyst electrode owing to the low overpotential for the H_2_ evolution reaction. The performance of a polymer electrolyte fuel cell is significantly increased by a good network of the perfluorosulfonate ionomers contacting with Pt nanoparticles (Uchida et al., 1995). The ionomer is also necessary to prepare the viscous catalyst ink for the Pt/CB catalyst layer with good bonding. Figure 9B shows the photocurrent density at the “gas | solid | gas” interfaces in the two-electrode system. The IPCE of the bare WO_3_/Ti fiber decreased from 8.2 to 3.2% by changing from the two-phase condition to the gas-phase condition. In contrast, the photocurrent densities of the ionomer-coated WO_3_/Ti fiber were the same for the “gas | solid | liquid” and the “gas | solid | gas” interfaces. As a result, the IPCE of the ionomer-coated WO_3_/Ti fiber was 180% higher than that of the bare WO_3_/Ti fiber electrode in the gas-phase PEC reaction.

**Figure 10:**
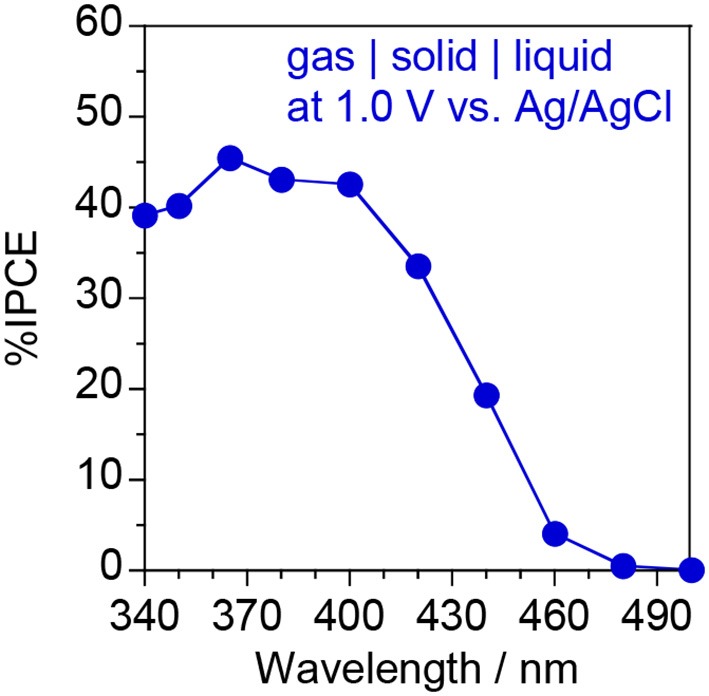
IPCE action spectrum of the ionomer-coated WO_3_/Ti fiber photoanode at 1.0 V vs. Ag/AgCl using the “gas | solid | liquid” interfaces shown in Figure 8A.

Figure 10 shows the IPCE action spectrum of the ionomer-coated WO_3_/Ti fiber electrode in the “gas | solid | liquid” interfaces. A visible-light response was observed at ~460 nm, which is consistent with the optical band gap of monoclinic WO_3_ nanoparticles (2.67 eV) estimated from the Tauc plot of the diffuse reflectance spectrum (Amano et al., 2017). In this study, the photocurrent response was investigated under blue light irradiation at 453 nm, which is very close to the threshold wavelength. This is the reason for the relatively low IPCE values under blue light. We found that the IPCE at 1.0 V vs. Ag/AgCl was higher than 40% under UV irradiation at wavelengths <400 nm. Moreover, the IPCE value was much higher than that of an ionomer-coated TiO_2_/Ti fiber (IPCE = 26% at 1.2 V under 365 nm UV irradiation) for gas-phase water oxidation (Amano et al., in press). To the best of our knowledge, this IPCE value is the highest among those reported for photoanodes in PEM-PEC systems.

## Discussion

We found that a proton-conducting ionomer coating enhanced the PEC oxidation of water in the gas phase, although this effect was not observed in aqueous media. The IPCE of the bare WO_3_/Ti fiber decreased from 28.1% in the liquid phase to 9.7–10.7% in the two-phase system and to 3.2% in the gas phase. This behavior indicates that an aqueous electrolyte is essential for PEC reactions that involve proton-coupled electron transfer. In the case of the gas-phase PEC reaction, the hydrated Nafion ionomer thin film plays the role of a solid electrolyte with good proton conductivity at room temperature. The ionomer coating also can absorb water molecules from the gas phase to enhance the hydration of the electrode surface. The Nafion ionomer membrane must be hydrated to maintain the high proton conductivity (Spurgeon and Lewis, 2011). The investigation of the PEC reaction in aqueous media revealed that the ionomer thin film slightly retards the transport of materials, which affects water adsorption and/or O_2_ desorption on the WO_3_ surface. However, the gas permeability of the thin film is sufficient to create a gas–electrolyte–solid triple phase boundary, where gaseous reactants and products are accessible.

Recently, we found that TiO_2_ electrodes without a Nafion ionomer coating exhibit negligible photocurrents for the PEC oxidation of gas-phase water vapor (Amano et al., in press). In contrast, the bare WO_3_/Ti fiber electrode exhibited a moderate photocurrent density during the gas-phase PEC reaction. This difference in behavior can be attributed to the proton conductivity of the oxide surfaces. The Nafion ionomer is a perfluorosulfonic acid with strong acidity, and WO_3_ is an acidic oxide with an isoelectric point at pH 1.5 (Anik and Cansizoglu, 2006). In contrast, TiO_2_ is a neutral oxide with an isoelectric point at pH 5–7 (Maeda and Domen, 2010). Thus, the acidic nature of the WO_3_ surface can slightly promote proton transport at room temperature. However, the proton conductivity of the bare electrode was not sufficient for the gas-phase PEC reaction. The Nafion ionomer coating enhanced the photocurrent density for the oxidation of water vapor to evolve O_2_. The FE of O_2_ evolution was enhanced to 80%, suggesting that the majority of the photogenerated holes were consumed by a four-electron reaction involving proton-coupled electron transfer.

The fabricated PEM-PEC cell shows a H_2_ evolution rate of ~1.0 μmol min^−1^ at 1.2 V under visible-light irradiation (λ = 453 nm, *I*_0_ = 6.8 mW cm^−2^). The rate of H_2_ evolution was much higher than that of the previously reported PEM-PEC systems (Stoll et al., 2016, 2017). The IPCE of 40% in the UV range was also the highest value in the gas-phase water photoelectrolysis reaction. This work highlights that the gas–electrolyte–solid triple phase boundary sustained by solid polymer electrolyte plays a significant role in enhancing the photocurrent in the absence of liquid water. To implement solar water splitting technologies, it is necessary to decrease the applied bias voltage and increase the IPCE in the visible light region.

In conclusion, we have successfully fabricated a PEC system consisting of WO_3_ nanoparticles for water vapor splitting under visible-light irradiation. A high-surface-area WO_3_/Ti fiber gas diffusion electrode was coated with a perfluorosulfonate electrolyte thin film to improve the proton conductivity. Under gas-phase conditions, the ionomer coating significantly enhanced the IPCE as well as the current efficiency of the O_2_ evolution reaction by four-electron oxidation of water vapor. The gas–electrolyte–solid triple phase boundary on the high-surface-area photoanode enhanced the extent of proton-coupled electron transfer between the photogenerated holes and the adsorbed water, which was fed from the gas phase. The concept provides insights into the features necessary for successful gas-phase operation, which is a promising approach for low-cost, large-scale H_2_ production under solar irradiation.

## Author contributions

FA designed and guided the study and wrote the paper; AS, HM, Y-MH, and KT carried out experiments and analyzed the data.

### Conflict of interest statement

The authors declare that the research was conducted in the absence of any commercial or financial relationships that could be construed as a potential conflict of interest.
